# Experimental Investigation of a Tubular Front Cavity for Wind Noise Suppression in MEMS Microphones of Mobile Devices

**DOI:** 10.3390/mi17030357

**Published:** 2026-03-14

**Authors:** Chengpu Sun, Shikun Wei, Bilong Liu

**Affiliations:** School of Mechanical & Automobile Engineering, Qingdao University of Technology, No. 777 Jialingjiang Road, Qingdao 266520, China; sunchp@foxmail.com (C.S.); weishikun02@163.com (S.W.)

**Keywords:** wind noise suppression, tubular front cavity, duct flow experiment, microphone

## Abstract

Wind-induced noise remains a critical engineering challenge for MEMS microphones in compact consumer electronics such as smartphones, where spatial constraints limit conventional noise control solutions. This study experimentally investigates the suppression of flow-induced wind noise by a straight tube serving as the front cavity of a microphone, using a precision measurement microphone for data acquisition. Controlled experiments were conducted in both a flow duct for parametric isolation and an anechoic chamber for real-world validation. Results demonstrate a strong diameter-dependent effect: for a 1 mm diameter, increasing tube length significantly reduces noise power spectral density and steepens high-frequency roll-off via enhanced internal viscous and thermal dissipation. This effect weakens for a 2 mm diameter and becomes negligible for a 3 mm diameter, where noise is dominated by external flow excitation at the tube inlet rather than internal propagation. Therefore, extending tube length is an effective noise control strategy only for small-diameter cavities. Furthermore, while increased wind speed and oblique incidence elevate PSD, a longer tube reduces this sensitivity. Because acoustic transmission loss—including potential effects like aperture diffraction and impedance mismatch—was not measured, any resulting improvement in the effective signal-to-noise ratio is strictly presented as a hypothesis requiring future electroacoustic validation. The consistent findings across both experimental environments provide clear design guidance: for compact MEMS microphone systems in portable devices, elongating the front cavity is a viable passive noise control method only when the cavity diameter is sufficiently small (<2 mm). This offers a practical, space-efficient alternative to traditional windscreen-based approaches in portable devices.

## 1. Introduction

Wind noise remains a critical challenge in audio communication systems, particularly for mobile devices such as smartphones and wearable gadgets. In outdoor environments, turbulent airflow around the device generates pressure fluctuations that degrade speech intelligibility and signal quality. Recent studies on wind noise monitoring [1] and hearing aids [2] highlight that even low-speed wind can saturate microphones and distort amplitude-modulated signals, underscoring the urgency of adaptive solutions. A parallel understanding of hydrodynamic instability mechanisms, such as the energy gradient theory proposed by Dou [3], emphasizes the role of energy gradients in triggering turbulence transitions, providing insights into turbulence suppression strategies for compact devices.

The physics of wind noise generation is rooted in the interaction between atmospheric turbulence and the device surface. Raspet et al. [4] demonstrated that wind noise in unscreened microphones is dominated by stagnation pressure fluctuations, which scale with the square of the incident wind velocity. Their work further highlighted the role of windscreen size in attenuating local pressure fluctuations, with larger screens (e.g., 100 cm diameter) achieving significant noise reduction through spatial averaging. However, such dimensions are impractical for handheld devices, where sub-centimeter profiles are required. Dou’s energy gradient theory [3] identifies the critical parameter Kmax—the ratio of transverse to streamwise energy gradients—as a universal predictor of turbulence onset, aligning with experimental observations in Poiseuille flows. This underscores the need to localize and suppress energy gradients in miniaturized systems.

Recent studies by Niu et al. [5] revealed that in wind tunnel environments, porous windscreens exhibit frequency-dependent noise suppression: turbulence-turbulence interaction dominates below 50 Hz, while stagnation pressure models fail to predict higher-frequency behavior due to suppressed inertial subranges. Zhao et al. [6] further demonstrated that spatial decorrelation is the key mechanism for noise reduction in porous windscreens, with optimal performance achieved when the windscreen diameter matches 2–4 times the turbulence wavelength. Kendrick et al. [1] extended this principle by proposing real-time wind noise detection algorithms to mitigate amplitude modulation errors caused by intermittent gusts, demonstrating a 50% reduction in metric bias. Similarly, Yu et al. [7] investigated ground-level wind noise and showed that porous foam layers could suppress turbulence–shear interactions near the surface. Their findings suggest that thin porous materials may offer a viable pathway for noise reduction in compact geometries, yet the trade-offs between material thickness, airflow resistance, and acoustic transparency remain unexplored.

Existing studies on windscreen design primarily focus on two mechanisms: spatial averaging of pressure fluctuations across the windscreen surface and dissipation of turbulent energy through porous materials. For large windscreens, the former dominates, as phase cancellation across the screen surface attenuates high-frequency noise. However, in miniaturized systems, the reduced correlation length of turbulence at high frequencies (typically >1 kHz) limits the effectiveness of spatial averaging. Instead, material-based dissipation—via fibrous or foam structures—becomes critical. An alternative or complementary approach involves recessing the microphone within a cavity to physically separate it from the turbulent boundary layer. This approach is particularly relevant for Micro-Electro-Mechanical Systems (MEMS) microphones, which are ubiquitous in mobile phones and wearables due to their small size, low power consumption, and high-volume manufacturability. The wind noise problem in these devices directly impacts voice call quality and audio recording fidelity. The fundamental theory of sound propagation in ducts, where pressure fluctuations can decay exponentially for cut-off modes within cavities of certain dimensions [8], provides a theoretical basis for this noise reduction strategy [9]. Experimental and applied research in wind-tunnel acoustics has systematically explored this concept, demonstrating that recessing microphones in cavities covered with low-impedance porous meshes (e.g., Kevlar) can significantly reduce turbulent boundary layer (TBL) noise—often termed “self-noise”—by up to 20 dB at lower frequencies [10,11]. Recent comprehensive evaluations, such as that by VanDercreek et al. [12], have quantified the impact of different cavity geometries (e.g., cylindrical hard-walled, conical melamine foam) on signal-to-noise ratio (SNR) and acoustic imaging accuracy in wind-tunnel arrays. Their work shows that optimized cavity designs, particularly those employing sound-absorbing materials, can attenuate TBL noise by up to 40 dB, significantly improving the detectability of acoustic signals.

Further developments in cavity-based noise reduction research, particularly using advanced simulation and deterministic modeling, provide deeper insights into the physical mechanisms. Mourão Bento et al. [13] employed Lattice Boltzmann Very Large Eddy Simulations (LBM-VLES) to study turbulent flow over axisymmetric cavities (cylindrical, countersunk, and conical) with and without porous covers. Their findings indicate that adding a countersink to a cylindrical cavity mitigates turbulent transport to the cavity bottom by shifting the recirculation pattern, while a porous cover nearly eliminates hydrodynamic pressure fluctuations, leaving a primarily acoustic pressure field inside. Proper Orthogonal Decomposition (POD) analysis in their study further revealed that for covered cavities, the pressure field at the bottom is dominated by cut-on acoustic modes, suggesting that acoustic modeling can effectively predict the propagation of TBL pressure fluctuations into such cavities. Building upon this, van Dercreek et al. [14] proposed a deterministic acoustic propagation model for cylindrical cavities based on duct acoustics theory. The model, which accounts for porous covers and both hard and soft walls, predicts the attenuation of TBL pressure fluctuations by evaluating the cut-off of higher-order acoustic modes within the cavity. Their results show reasonable agreement with experimental data and highlight that the primary attenuation mechanism for small cavities is the exponential decay of cut-off modes with depth. Complementing these modeling efforts, VanDercreek et al. [15] conducted an extensive experimental campaign using a Design of Experiments (DOE) approach to systematically evaluate the effects of cavity depth, area, mesh covering, and area change on TBL spectral energy and SNR. Their study, which employed a Generalized Additive Model (GAM) for data analysis, quantified that a mesh cover reduces spectral energy by approximately 8 dB and that reducing the cavity area from the aperture to the base further decreases the measured TBL noise. The study confirmed that the propagation of pressure fluctuations within such cavities aligns with the theoretical framework of cut-off acoustic modes.

Zakis et al. [2] revealed that microphone saturation at 12 m/s flattens wind noise spectra, masking speech across all frequencies and necessitating adaptive gain control in hearing aids—a challenge equally critical for mobile devices. While Zhao et al. [16] demonstrated that even 1 inch foam layers could align measurements with turbulence-shear interaction predictions, such thicknesses exceed the form factor constraints of mobile devices. Niu et al. [5] further emphasized that ultra-thin windscreens must address the transition between energy-containing and dissipation ranges, where conventional von Karman-type turbulence models are invalid due to low Reynolds numbers. Recent advances by Zhao et al. [17] established a pressure spectrum model for small Reynolds number flows, showing that wind noise spectra in compact devices exhibit flat low-frequency plateaus and exponential high-frequency decay, bypassing the traditional k^−7/3^ scaling. Moreover, the interaction between ultra-thin porous structures and boundary layer turbulence remains poorly understood, particularly in the context of omnidirectional airflow around irregular device shapes.

A key limitation of current models is their reliance on static, homogeneous turbulence spectra. Mobile devices operate in highly dynamic environments where wind speed, angle of incidence, and turbulence scales vary rapidly. For example, Raspet et al. [4] emphasized the inertial subrange (k^−5/3^ scaling) as the primary regime for windscreen performance, yet handheld devices often experience turbulence transitions between the source region (k^−5/3^ scaling) and the inertial subrange due to their small characteristic lengths. This necessitates adaptive designs that balance low-frequency stagnation pressure suppression and high-frequency turbulence dissipation. Recent advances in generalized porous media modeling, such as the non-Darcian framework proposed by Nithiarasu et al. [18], provide insights into variable porosity effects and channeling phenomena, which are critical for optimizing microscale windscreens. Furthermore, computational studies by Xu et al. [19] revealed that windscreen shape and flow resistivity significantly influence self-noise generation, with horizontal elliptical profiles outperforming vertical configurations by minimizing wake turbulence.

Current research on microphone wind noise mitigation predominantly focuses on windscreen optimization or, in aerodynamic testing contexts, on recessed cavities for high-speed flow. However, the implementation of conventional windscreen designs faces significant challenges in portable communication devices (e.g., smartphones) equipped with MEMS microphones due to stringent spatial constraints. While prior studies on cavity-based noise reduction [9,10,11,12,13,14,15] have validated the principle and provided valuable design guidelines for specific applications like wind-tunnel arrays, they are primarily situated in the context of higher flow speeds (e.g., 30–70 m/s) and focus on turbulent boundary layer noise suppression for aerodynamic acoustic measurements. Moreover, those studies typically employ cavities with diameters significantly larger than those considered here, where spatial averaging and cut-off modes dominate. In contrast, the present work investigates small-diameter cavities (≤3 mm) in low-speed flows (4–8 m/s), where viscous and thermal boundary-layer dissipation is the primary attenuation mechanism—a regime directly relevant to MEMS microphones in portable electronics. Our results demonstrate that for a small-diameter cavity (d = 1 mm), increasing the cavity length leads to a pronounced and systematic reduction in the PSD across mid-to-high frequencies, characterized by a progressively steeper spectral roll-off. This observed attenuation trend is significant; it is comparable in effect to the noise reduction achieved by optimized cavity designs in higher-speed flows, such as the up to 20 dB reduction in turbulent boundary layer noise reported by Jaeger et al. [10] and VanDerreck et al. [12]. However, our work achieves this within a simplified, space-efficient tubular geometry under low-speed conditions, highlighting its direct applicability to MEMS microphones in portable electronics.

This study therefore investigates the influence of key geometric parameters in a simple, straight-tube front cavity—specifically its length, diameter, and wind incidence angle—on wind noise generation in scenarios mimicking smartphone MEMS microphone environments. To fundamentally understand the geometric influence of the front cavity, a well-controlled experimental model was constructed using a precision measurement microphone to ensure high-fidelity acquisition of subtle intra-cavity pressure fluctuations. The results elucidate the dominant role of cavity diameter and the efficacy of length extension under specific conditions, establishing a foundational principle for developing space-efficient front-end acoustical structures for MEMS microphones in portable electronics. Experiments were conducted in both a flow duct and an anechoic chamber to isolate and analyze the geometric effects.

## 2. Duct Flow Experimental

To isolate and quantify the influence of key parameters—the tubular front cavity diameter, length, and wind incidence angle—on flow-induced noise generation, a controlled duct flow experiment was first conducted. This environment allows for precise regulation of flow velocity and turbulence characteristics, enabling a fundamental investigation into the underlying flow-cavity interaction mechanisms. Subsequently, to validate the generality of the findings under conditions more representative of real-world applications (e.g., open-field, non-uniform flow), parallel experiments were performed in an anechoic chamber using a free-field wind source. This two-pronged experimental approach ensures that the identified design principles are robust and not artifacts of a specific test configuration.

### 2.1. Experimental Setup and Sample

**Figure 1:**
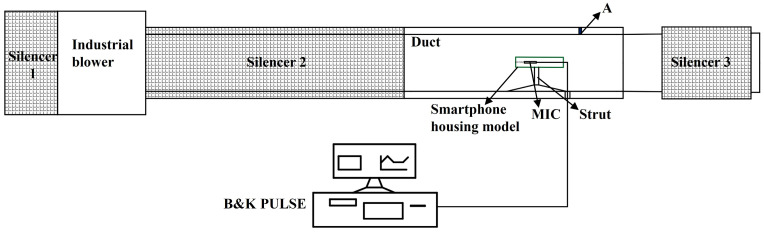
Schematic diagram of the experimental system. Point A denotes the measurement location for the wall fluctuating pressure.

A centrifugal blower-ducted experimental system was designed and constructed to achieve precise airflow velocity control, as illustrated in Figure 1, comprising a modular airflow generation and acoustic measurement assembly designed to simulate realistic smartphone microphone exposure under controlled wind conditions. Key components include:

Airflow Source: The centrifugal industrial blower (Type: YKL9-26-4.5A-X, Zhejiang Keli Fan Co., Ltd., Shaoxing (Shangyu Economic Development Zone), Zhejiang Province, China) employs a variable frequency drive (VFD) system (Type: JCDK2.2, Shaoxing Shangyu Jinzong Electrical Control System Co., Ltd., Shaoxing (Shangyu District), Zhejiang Province, China) with a frequency modulation range of 10–50 Hz to precisely regulate airflow velocity, achieving controlled flow rate between 0.13 m^3^/s and 0.67 m^3^/s. A three-stage silencer configuration—comprising two perforated-plate silencers in series upstream and a third unit downstream—effectively suppresses blower-generated broadband noise, achieving a background sound pressure level (SPL) below 20 dB@1 kHz at the test section.

Duct: The duct with smooth walls consists of a rectangular polymethyl methacrylate (PMMA) duct with an internal cross-section of 250 mm × 250 mm and a length of 1000 mm, featuring a 12 mm wall thickness. This optically transparent enclosure accommodates a smartphone housing model positioned centrally via a custom-designed strut mounting system.

Acquisition System: A Bruel & Kjær PULSE (Type: 3160-A-042, (B&K), Nærum, Denmark) system with 1/4 inch microphones (Type: MPA401, BSWA Technology Co., Ltd., Beijing, China) samples acoustic data. A handheld digital manometer, operating on the pitot tube principle (model KLH-5000, Suzhou Kelihua Electronics Co., Ltd., Suzhou, China), was used to measure the wind speed (maintained for 10 s prior to data acquisition).

The industrial blower is connected to silencer 2 via a transition duct with variable diameters. Downstream, silencer 3 is connected to the duct outlet using a matched-diameter duct, ensuring geometric continuity (tolerance: ±0.5 mm inner diameter).

To ensure the reliability of the experimental data, each cavity configuration was measured at least six times under identical flow conditions. The power spectral density (PSD) curves presented in this work are the ensemble averages of these repeated measurements, which effectively suppress random fluctuations and highlight the deterministic trends associated with geometric and flow parameters. Statistical analysis revealed that the standard deviation of PSD levels across the six repetitions remained below 1.5 dB throughout the frequency range of 20 Hz to 20 kHz, confirming that the observed differences between configurations are statistically significant. Prior to each test session, the measurement microphones were calibrated using a BSWA CA111 Sound Calibrator (94 dB and 114 dB at 1000 Hz, BSWA Technology Co., Ltd., Beijing, China, Class 1 per IEC 60942:2003 [20]). These procedures collectively validate the robustness and reproducibility of the reported results.

**Figure 2:**
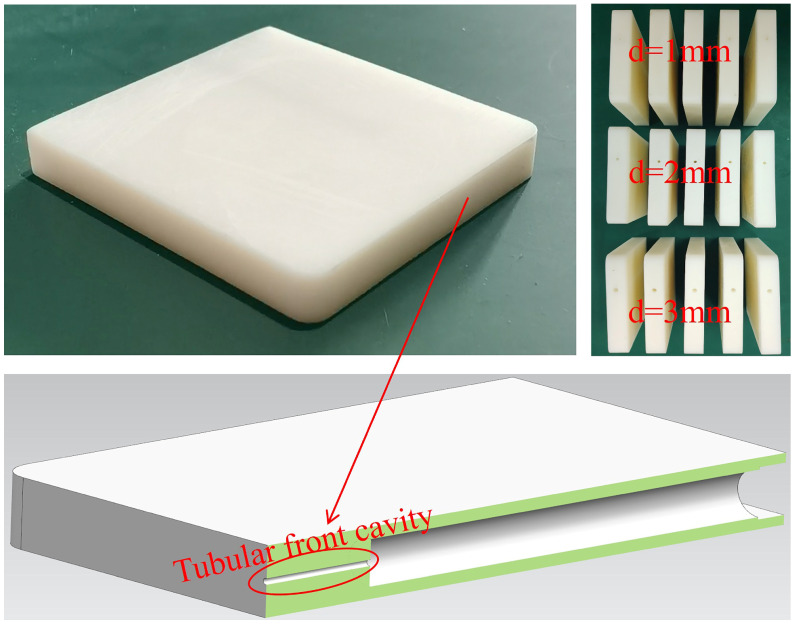
Smartphone housing model and Schematic diagram of its cross-section.

The smartphone housing model (L × W × H, 80 mm × 73 mm × 11 mm) is rigidly centered in the duct via struts, with its cavity inlet directly facing the flow at adjustable angles. This configuration permits flow-induced noise characterization for internal microphones across the variable diameter and length of the tubular front cavity, as well as inflow angles. A series of tubular front cavity samples with varied diameters and lengths was custom-fabricated (tolerance: ±0.1 mm, PMMA material) for parametric analysis, as schematically illustrated in Figure 2. The experimental matrix comprises 15 samples covering three diameters (d = 1/2/3 mm), each with five lengths (L = 1/4/8/16/24 mm). The smartphone housing model and the integrated tubular cavities were fabricated using 3D printing with 8228Pro resin (ABS-like Photopolymer Resin), a rigid and non-porous photopolymer. After printing, the inner walls of the cavities were carefully polished to achieve a smooth surface finish. This process ensures that the cavity walls are acoustically hard and rigid, with no porous or sound-absorbing characteristics.

Prior to the experiments, the pressure spectrum on the duct wall was measured and compared with simulation results to validate whether turbulent fluctuating pressure could be generated as anticipated. The measurement point on the pipe wall is indicated as Point A. A microphone was flush-mounted at Point A on the sidewall. To prevent spatial averaging of the fluctuating pressure by the microphone diaphragm, the microphone was not directly exposed to the pipe flow. Instead, a 1 mm diameter, 1 mm deep pinhole was positioned directly in front of the microphone diaphragm center on the wall at Point A.

### 2.2. Measured and Predicted Pressure Spectrum at the Wall of the Duct

**Figure 3:**
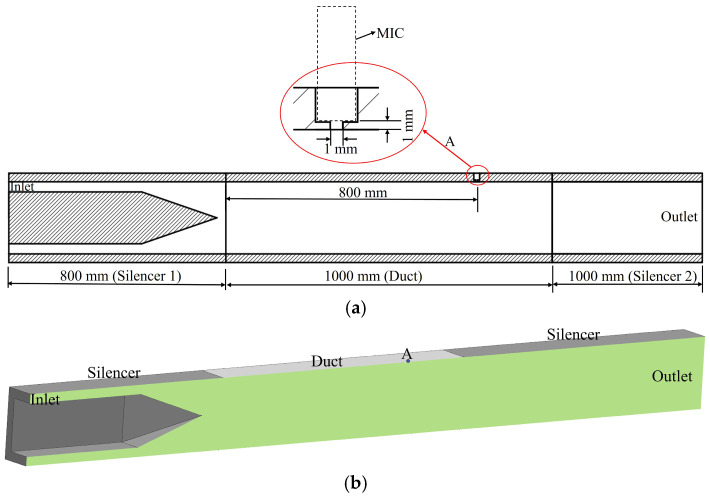
CFD geometry. (**a**) Duct cross-section schematic diagram. (**b**) Fluid domain geometric model.

To verify whether the experimental setup could generate the anticipated turbulent pressure fluctuations within the duct, the wall pressure spectrum was predicted using computational fluid dynamics (CFD) and compared with the measurements at Point A. Considering both computational efficiency and result accuracy, the computational domain for the CFD simulations comprised the duct section from the midsection of Silencer 2 to the outlet of Silencer 3 (Figure 3). Specifically, this included the 800 mm rear portion of Silencer 2, the full 1000 mm duct, and the complete 1000 mm length of Silencer 3.

The CFD simulations were performed using ANSYS Fluent (version: 2024R1) with a two-step strategy to ensure both numerical stability and physical accuracy. First, a steady RANS simulation employing the SST k-ω turbulence model was conducted to obtain a fully converged mean flow field. This steady solution was then used as the initial condition for a transient Large Eddy Simulation (LES). The LES was run for a sufficiently long physical time to allow the flow to develop into a statistically stationary turbulent state, effectively eliminating any residual influence from the RANS initialization.

A high-quality mesh was generated with particular attention to near-wall resolution. The grid was carefully refined to maintain the dimensionless wall distance y^+^ below unity (y^+^ ≈ 1) for all simulated conditions, satisfying the stringent requirements of wall-resolved LES. In the steady SST k-ω simulation, convergence was assumed when residuals for all governing equations fell below 10^−6^. For the transient LES, convergence within each time step was ensured by performing sub-iterations until residuals dropped by at least one order of magnitude, thereby maintaining numerical stability and temporal accuracy.

**Figure 4:**
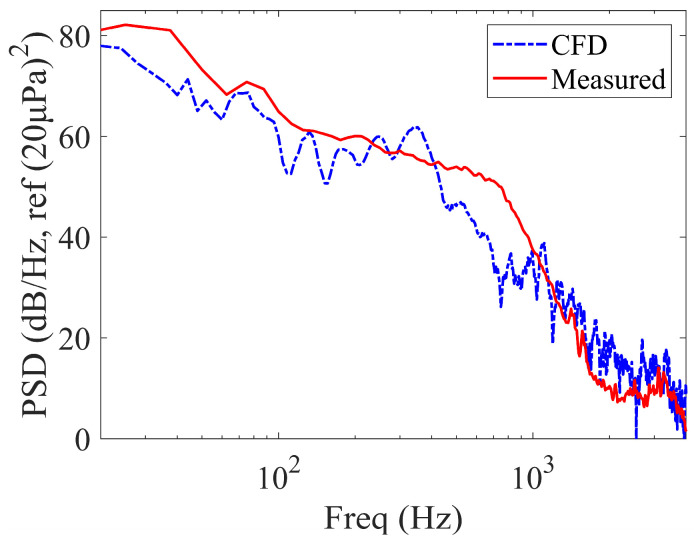
Comparison of Measured and CFD-Simulated Wall Pressure Fluctuation PSD of the duct. Wind speed at the duct: 6 m/s.

A traditional grid independence study was not performed for the transient LES, as the computational cost of running multiple finely resolved LES cases would be prohibitive. Instead, the mesh was constructed strictly adhering to best-practice guidelines for wall-resolved LES (y^+^ ≈ 1). Crucially, the numerical model was configured with physical dimensions and boundary conditions identical to those of the experimental setup. As shown in Figure 4, the excellent agreement between the LES predictions and experimental measurements provides the most direct and robust validation possible—demonstrating that the current mesh resolution and numerical scheme are fully capable of capturing the relevant physical phenomena.

Based on the fluid domain model shown in Figure 3a, the outer end face of the sidewall orifice (diameter: 1 mm) was designated as the pressure measurement point (see the fluid domain in Figure 3b). In the CFD simulation, an inlet velocity of 12 m/s was defined to match the experimental condition (resulting in an average velocity of 6 m/s at the duct). The walls were treated as no-slip boundaries. The area-averaged static pressure at the 1 mm orifice (Point A) was then extracted for analysis. A comparison of the power spectral density (PSD) obtained from experiments and CFD simulations is presented in Figure 4.

As shown in Figure 4, the measured and simulated PSD of turbulent pressure fluctuations on the duct wall exhibit good agreement. The spectrum displays a plateau trend within the energy-containing range (≤40 Hz). The frequency band from 40 Hz to 800 Hz corresponds to the inertial subrange, while frequencies > 800 Hz fall within the dissipation range. Beyond 2 kHz, the curve levels off, indicating the background noise floor. The measured and simulated results collectively demonstrate that the experimental apparatus employed in this study successfully generates the required turbulent pressure fluctuations within the duct.

### 2.3. Experimental Results and Discussions

To investigate the mechanisms governing flow-induced noise in microphones with tubular front cavities, a series of wind tunnel experiments was conducted by varying both structural and non-structural parameters. The structural parameters include the diameter and length of the tubular front cavity, while the non-structural parameters involve the wind speed and wind incidence angle. By independently controlling these variables, the experimental results provide insight into the relative contributions of internal acoustic dissipation and external flow excitation to the overall noise characteristics.

**Figure 5:**
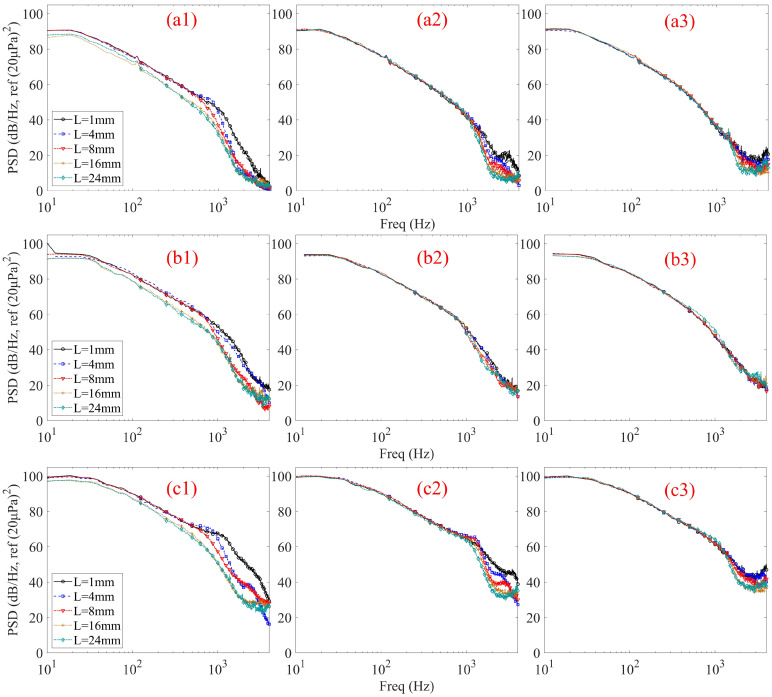
Flow-induced noise PSD of different tubular front cavity lengths at 0° incidence angles. Subplots (**a1**–**a3**), (**b1**–**b3**), and (**c1**–**c3**) correspond to wind speeds of 4, 6, and 8 m/s, respectively; within each speed group, diameters of the tubular front cavity are 1, 2, and 3 mm from left to right.

Figure 5 illustrates the flow-induced noise PSD for different tubular front cavity lengths under three cavity diameters at a zero incidence angle and three wind speeds. At the lower wind speed of 4 m/s (Figure 5(a1–a3)), the influence of the tubular front cavity length exhibits a clear dependence on the cavity diameter. For the 1 mm diameter case (Figure 5(a1)), increasing the tubular front cavity length leads to a noticeable reduction in PSD in the mid- to high-frequency range, particularly above several hundred hertz, accompanied by an earlier spectral roll-off. This indicates that, for small diameters, the internal duct length plays a significant role in attenuating flow-induced pressure fluctuations even under relatively weak turbulent excitation. When the cavity diameter increases to 2 mm (Figure 5(a2)), the effect of the tubular front cavity length becomes markedly weaker. The PSD curves corresponding to different cavity lengths almost collapse over the entire frequency range, with only minor deviations at high frequencies. For the 3 mm diameter case (Figure 5(a3)), the PSD spectra for all cavity lengths are nearly indistinguishable, indicating that variations in the tubular front cavity length no longer exert a meaningful influence on the flow-induced noise characteristics. At a wind speed of 6 m/s (Figure 5(b1–b3)), similar trends are observed, while the overall PSD levels increase due to the stronger turbulent excitation. For the 1 mm diameter case (Figure 5(b1)), a clear dependence on the tubular front cavity length persists across the mid- to high-frequency range. As the cavity length increases, the PSD level shows a progressive reduction, particularly above several hundred hertz, accompanied by an earlier and steeper spectral roll-off. This behavior suggests enhanced viscous and thermal boundary-layer losses, as well as increased attenuation of flow-induced pressure fluctuations along the tubular front cavity wall. In contrast, for the 2 mm diameter (Figure 5(b2)), the influence of cavity length is significantly reduced, and for the 3 mm diameter (Figure 5(b3)), the PSD spectra corresponding to different lengths are almost indistinguishable. At the highest wind speed of 8 m/s (Figure 5(c1–c3)), the same diameter-dependent behavior is consistently observed. For the 1 mm diameter case (Figure 5(c1)), increasing the tubular front cavity length remains effective in suppressing mid- and high-frequency noise, despite the intensified turbulent fluctuations. However, for the 2 mm and 3 mm diameter cases (Figure 5(c2,c3)), the effect of cavity length is again minimal, demonstrating that the insensitivity to cavity length is robust with respect to changes in flow velocity once the cavity diameter becomes sufficiently large.

Collectively, the data in Figure 5 reveal a clear and consistent pattern across all tested flow velocities: the influence of the tubular front cavity length on noise suppression exhibits a strong dependence on the tubular front cavity diameter. The effectiveness of a long, slender cavity (e.g., 1 mm diameter) in suppressing wind noise aligns with the underlying principle of turbulence energy dissipation. This can be interpreted through the lens of the energy gradient theory [3]: by increasing the flow path and surface area, the elongated cavity enhances viscous and thermal dissipation, effectively diminishing the transverse energy gradients within the flow that are critical for sustaining turbulence and pressure fluctuations. This mechanism shares a conceptual synergy with the noise suppression observed in porous windscreens [5,7,8], where the material structure dissipates turbulent kinetic energy. Consequently, while increasing cavity length is a potent geometric strategy for small-diameter cavities across a wide range of flow velocities, its utility diminishes rapidly as the diameter increases. For larger diameters, where internal dissipation becomes negligible relative to external flow excitation, noise reduction efforts must shift focus toward optimizing inlet geometry or implementing external flow control.

The observed diameter-dependent noise suppression can be interpreted using duct acoustics theory. For a circular tube of diameter d, the cut-off frequency for the first higher-order mode (1,1) is given by *f_c_* = 1.84c_0_/(πd), where c_0_ is the speed of sound. For d = 1 mm, *f_c_* ≈ 200 kHz, well above the frequency range of interest, indicating that plane wave propagation dominates. In this regime, the attenuation due to viscous and thermal losses at the boundary layer is governed by the boundary layer thickness, which scales with $\sqrt{\nu /\omega }$ (where ν is the kinematic viscosity and ω is the angular frequency). This relationship is a cornerstone of classical acoustics (see [21]) and underpins the enhanced dissipation with increasing tube length. For d = 3 mm, however, the boundary layer thickness is small relative to the diameter, and internal losses are negligible. Instead, external flow excitation at the inlet dominates, consistent with the spectral collapse observed in Figure 5. This physical interpretation—invoking cut-off frequencies, plane wave dominance, and boundary layer dissipation—is fundamentally consistent with the theoretical framework detailed by Rienstra and Hirschberg [8] and provides a robust basis for understanding the experimental trends.

It is important to distinguish between the attenuation of flow-induced pressure fluctuations and that of acoustic signals. While idealized plane-wave theory suggests minor viscothermal losses for acoustic signals in such short tubes, this simplification neglects aperture diffraction and impedance mismatch at the cavity interfaces, which inherently introduce acoustic Transmission Loss (TL). Measuring flow-induced noise (turbulent boundary layer pressure fluctuations) in miniature geometries is experimentally challenging and exceedingly difficult to simulate accurately using current CFD tools, making it the primary empirical focus of this study. Conversely, acoustic TL can be effectively modeled using finite element tools (e.g., COMSOL 6.3). Because a comparative experiment isolating acoustic TL under simultaneous flow was not conducted in this study, any resulting improvement in the operational Signal-to-Noise Ratio (SNR) remains a hypothesis. The preservation of acoustic transparency cannot be definitively claimed here and serves as a critical area for future dual-excitation studies.

**Figure 6:**
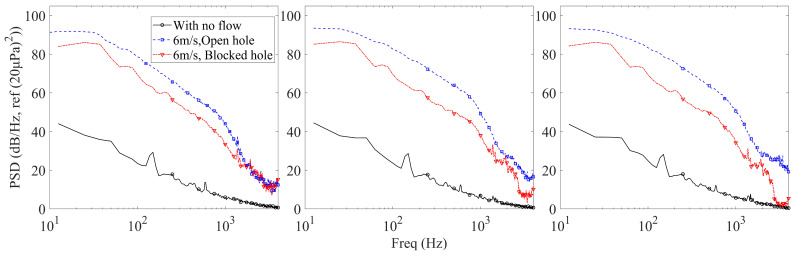
Power spectral density (PSD) comparison under three test conditions for the tubular front cavity with length 24 mm and diameters of 1 mm (**left**), 2 mm (**middle**), and 3 mm (**right**).

To clarify the origin of the high-frequency spectral plateau, additional baseline measurements were performed and are shown in Figure 6. For each front-cavity diameter (1–3 mm, length 24 mm), three conditions were compared: no flow, 6 m/s with an open hole, and 6 m/s with a blocked hole.

Under the no-flow condition, the measured spectrum represents the inherent noise floor of the microphone and acquisition system. When the pickup hole is blocked while maintaining a 6 m/s external flow, the microphone is acoustically isolated from direct aerodynamic excitation. In this case, the measured spectrum mainly reflects the intrinsic system noise together with any structure-borne vibration transmitted through the housing.

As shown in Figure 6, above approximately 2 kHz, the PSD curves under different operating conditions converge toward a relatively flat plateau. Importantly, this plateau level is close to that measured under the no-flow condition, indicating that the measurement in this frequency range is primarily noise-floor-limited rather than flow-excitation-dominated. In contrast, in the low- and mid-frequency range (below approximately 1–2 kHz), the open-hole condition exhibits significantly higher PSD levels than both the no-flow and blocked-hole conditions, confirming that aerodynamic pressure fluctuations dominate in this frequency range.

The variation in the high-frequency plateau level among different cavity diameters does not imply a change in intrinsic microphone noise, but rather reflects differences in acoustic transmission characteristics of the front cavity. The front cavity behaves as an acoustic impedance element whose geometry influences high-frequency attenuation and reflection. Changes in diameter modify the acoustic resistance and reactive components of the cavity, thereby altering the effective coupling between the microphone diaphragm and both internal electrical noise and any residual external disturbances. Consequently, slight shifts in the measured plateau level can occur due to geometry-dependent transmission effects and measurement system sensitivity limits, even though the intrinsic microphone noise remains unchanged.

These additional baseline comparisons therefore demonstrate that the spectral plateau above 2 kHz is predominantly governed by the inherent noise characteristics of the measurement system, while the wind-induced noise contribution is mainly concentrated in the low- and mid-frequency bands.

**Figure 7:**
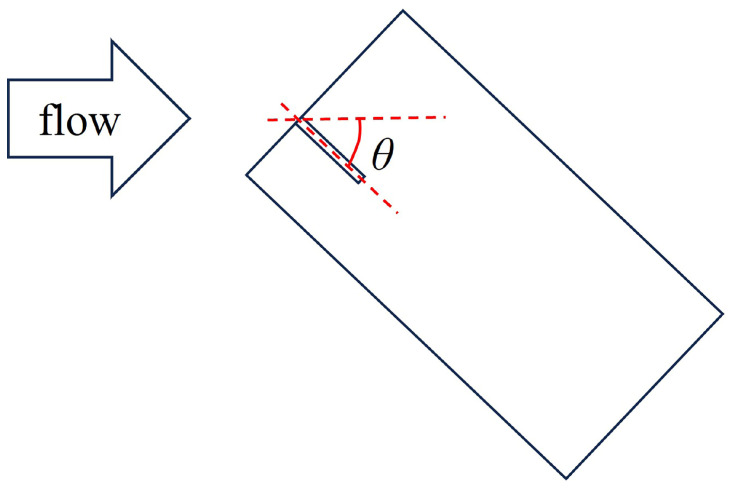
Schematic diagram of smartphone housing model orientation for wind incidence angles *θ*.

**Figure 8:**
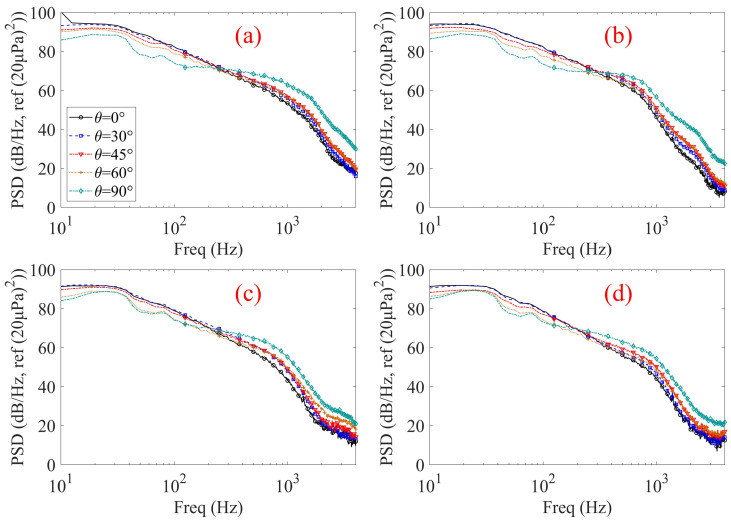
Flow-induced noise PSD of the samples at 6 m/s wind speed under different wind incidence angles measured in the duct flow experiment. (**a**) d = 1 mm, L = 1 mm; (**b**) d = 1 mm, L = 8 mm; (**c**) d = 1 mm, L = 16 mm; (**d**) d = 1 mm, L = 24 mm.

While the results presented above focus on axial inflow conditions (*θ* = 0°), practical operating environments for portable electronic devices are rarely characterized by purely axial wind exposure. In realistic scenarios, wind can impinge on the microphone inlet from arbitrary directions, leading to additional flow separation, shear-layer distortion, and enhanced vortex shedding at the cavity entrance. Therefore, to further elucidate the role of non-structural flow parameters, the influence of wind incidence angle on flow-induced noise is examined. Flow-induced noise data were collected under five wind incidence angles: 0°, 30°, 45°, 60°, and 90° (a schematic of angle *θ* is provided in Figure 7). Additionally, measurements were performed at three different wind speeds, corresponding to average velocities at the measurement point: 4 m/s, 6 m/s, and 8 m/s, as shown in Figure 8.

The measured PSD results shown in Figure 8 demonstrate that the flow-induced noise at the internal microphone position is strongly influenced by both the wind incidence angle and the geometric parameters of the microphone front cavity inlet. For all inlet configurations shown in Figure 8a–d, the PSD exhibits a pronounced low-frequency dominance and a monotonic decay with increasing frequency, which is characteristic of turbulence-induced pressure fluctuations.

For the shortest cavity configuration (L = 1 mm, Figure 8a), a strong dependence on wind incidence angle is observed. As the wind incidence angle increases from axial flow (*θ* = 0°) to transverse flow (*θ* = 90°), the PSD level rises progressively, particularly in the mid-to-high frequency range. This behavior can be attributed to enhanced unsteady shear-layer interaction and vortex shedding at the cavity inlet opening under oblique and transverse inflow conditions, which promotes stronger turbulence–acoustic coupling and more efficient transmission of external pressure fluctuations into the tubular front cavity. When the tubular front cavity length is increased to 8 mm (Figure 8b), the overall spectral shape remains similar; however, the PSD levels are systematically reduced and the sensitivity to wind incidence angle becomes weaker. This indicates that increasing the cavity length begins to suppress the transmission of flow-induced pressure fluctuations, especially at higher frequencies. A more pronounced attenuation effect is observed for the 16 mm cavity (Figure 8c). Compared with the shorter configurations, the PSD levels are further reduced across the measured frequency range, and the separation between curves corresponding to different incidence angles becomes noticeably smaller. This suggests that the extended cylindrical duct acts as an effective passive acoustic filter, where viscous and thermal boundary-layer losses, together with multiple internal reflections, progressively dissipate incoming turbulent pressure fluctuations. For the longest cavity configuration (L = 24 mm, Figure 8d), the flow-induced noise exhibits the weakest dependence on wind incidence angle. The PSD curves corresponding to different angles are closely clustered, indicating that sufficiently long tubular front cavities can effectively decouple the internal acoustic field from the external flow directionality. Under this condition, high-frequency components are strongly attenuated, and the directional dependence imposed by the external flow is largely suppressed before reaching the internal microphone location.

Overall, these results highlight the critical role of microphone inlet geometry in mitigating flow-induced noise and demonstrate that increasing the tubular front cavity length is an effective design strategy for reducing flow-induced disturbances in compact electronic devices, particularly under realistic, non-axial wind exposure conditions.

The above theoretical analysis indicates that, for the small-diameter elongated cavities considered in this study, the attenuation of acoustic signals is negligible compared to the suppression of wind-induced pressure fluctuations. Consequently, an improvement in the signal-to-noise ratio (SNR) is expected in windy environments. However, direct experimental validation of microphone sensitivity and SNR under combined acoustic and flow excitation lies beyond the scope of the present work, which focuses on the fundamental mechanisms of wind noise suppression.

## 3. Anechoic Chamber Experiment

**Figure 9:**
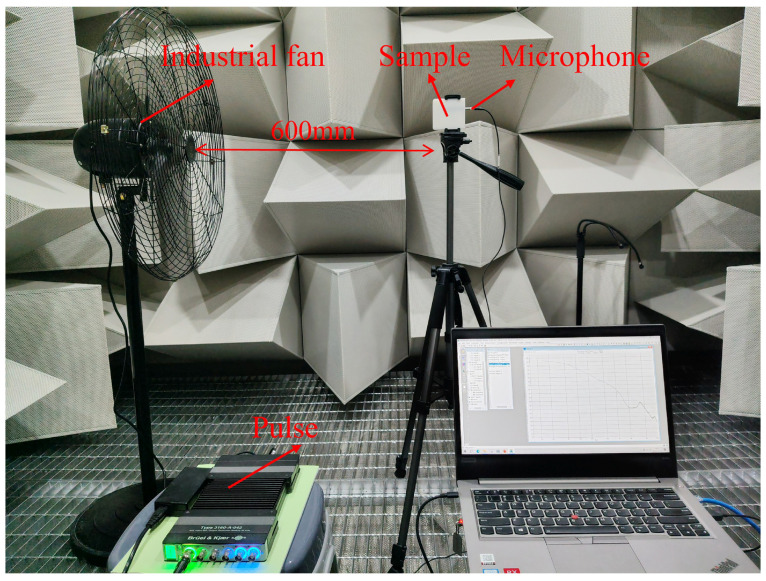
Wind noise test setup in an anechoic chamber.

**Figure 10:**
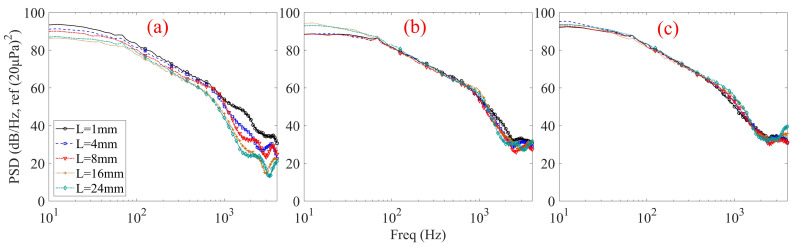
Wind noise PSD of all samples measured in the anechoic chamber at 6 m/s and 0° incidence angles. (**a**) d = 1 mm; (**b**) d = 2 mm; (**c**) d = 3 mm.

**Figure 11:**
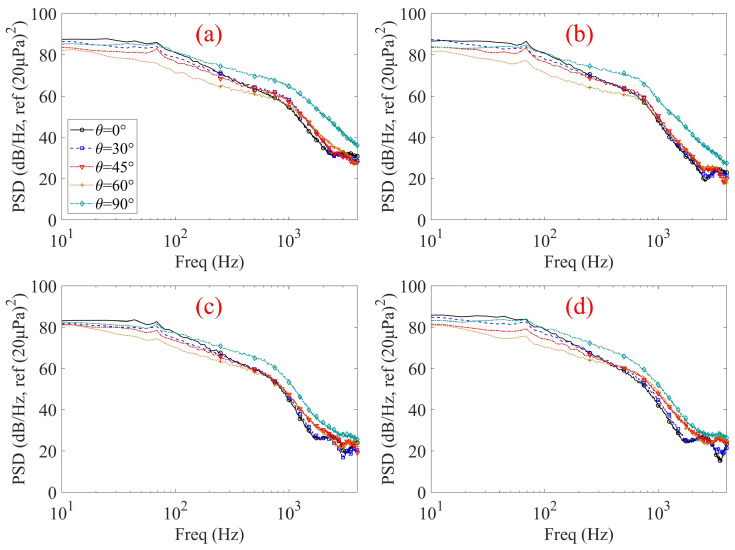
Flow-induced noise PSD of the samples at 6 m/s wind speed under different wind incidence angles measured in the anechoic chamber. (**a**) d = 1 mm, L = 1 mm; (**b**) d = 1 mm, L = 8 mm; (**c**) d = 1 mm, L = 16 mm; (**d**) d = 1 mm, L = 24 mm.

Since the environment inside the duct significantly differs from real-world mobile application scenarios, parallel tests were conducted on the sample within an anechoic chamber, as shown in Figure 9. An industrial fan (maximum power: 200 W) served as the wind source, positioned 600 mm away from the sample. All other instrumentation and setup remained the same as those used in the duct flow experiment. As the effects of wind speed and incidence angle are independent of the cavity within the samples, the tests in the anechoic chamber focused on evaluating the capability of the tubular front cavity to suppress wind noise in a relatively open environment. Consequently, this section emphasizes a comparative analysis of the impact of the tubular front cavity on wind noise. The PSD measurement results for all samples are presented in Figure 10 and Figure 11.

Consistent with the duct flow experiment, the tests conducted in the anechoic chamber fully corroborate the diameter-dependent effect of the tubular front cavity length on wind noise suppression. As shown in Figure 10, the capability of the tubular front cavity to attenuate noise diminishes sharply with increasing diameter. For the 1 mm diameter (Figure 10a), increasing the tubular front cavity length still leads to a clear reduction in PSD and a steeper high-frequency roll-off, confirming that internal viscous and thermal dissipation remain effective in an open environment for slender geometries. This effect becomes markedly weaker for the 2 mm diameter (Figure 10b), where PSD curves for different lengths largely overlap. Finally, for the 3 mm diameter (Figure 10c), the spectra are nearly indistinguishable across all lengths, demonstrating that the influence of the tubular front cavity length is negligible for larger diameters. The agreement between the two experimental setups confirms that the transition from internal-dissipation-dominated to external-excitation-dominated noise generation with increasing diameter is an intrinsic property of the tubular front cavity structure and is independent of the experimental environment.

Figure 11 shows the flow-induced noise PSD of four samples with identical tubular front cavity diameter (d = 1 mm) but different cavity lengths under a wind speed of 6 m/s and varying wind incidence angles, measured in an anechoic chamber. For all configurations, the PSD spectra exhibit a pronounced low-frequency dominance and a gradual decay with increasing frequency, consistent with the turbulence-induced noise characteristics observed in the ducted wind experiments shown in Figure 8.

For the shortest cavity configuration (L = 1 mm, Figure 11a), the flow-induced noise exhibits a strong dependence on wind incidence angle. The PSD levels increase noticeably as the inflow direction shifts from axial to oblique and transverse conditions, with the highest noise levels occurring under transverse inflow (*θ* = 90°), particularly in the mid-to-high frequency range. This behavior indicates that non-axial inflow, especially transverse wind exposure, can strongly enhance inlet shear-layer instability and vortex shedding, thereby promoting more efficient transmission of flow-induced pressure fluctuations into the cavity. In this case, the most unfavorable noise condition corresponds to transverse rather than intermediate inflow angles. When the tubular front cavity length is increased to 8 mm (Figure 11b), the overall spectral shape remains similar; however, both the absolute PSD levels and the separation between different incidence angles are reduced compared with the shortest cavity case. Although transverse inflow (*θ* = 90°) still yields relatively higher PSD levels, the contrast among different inflow directions becomes less pronounced, indicating a partial suppression of angular sensitivity. A further reduction in angular sensitivity is observed for the 16 mm cavity (Figure 11c). The PSD curves corresponding to different wind incidence angles become closely spaced over most of the frequency range, with only slight residual differences in the mid-frequency region, suggesting that the influence of inflow direction is increasingly mitigated as the tubular front cavity length increases. For the longest cavity configuration (L = 24 mm, Figure 11d), the PSD spectra under different wind incidence angles are highly clustered across the entire frequency range, demonstrating that the directional dependence of flow-induced noise is effectively suppressed. Under this condition, the internal acoustic response is largely decoupled from the external flow direction before the pressure fluctuations reach the internal microphone position.

The progressive weakening of angular sensitivity with increasing tubular front cavity length observed in Figure 11 is in good qualitative agreement with the ducted wind test results shown in Figure 8. In both experimental environments, short cavities exhibit pronounced sensitivity to non-axial and transverse inflow, whereas sufficiently long cavities effectively mitigate the influence of wind incidence angle through cumulative viscous and thermal dissipation and internal acoustic attenuation. Despite differences in flow generation mechanisms and test configurations, the consistent trends observed across the two datasets indicate that the dominant noise suppression mechanisms are primarily governed by the tubular front cavity geometry rather than the external test environment. Building on this robustness, the overall results further demonstrate a strong diameter-dependent sensitivity to cavity length: extending the tubular front cavity length is highly effective in suppressing high-frequency wind noise and angular dependence for slender cavities with small diameters, but its effectiveness diminishes rapidly as the diameter increases.

## 4. Conclusions

This study investigated the influence of the tubular front cavity on wind-induced noise in compact microphone systems through complementary duct flow and anechoic chamber experiments. The results demonstrate that the noise suppression capability of a tubular front cavity is governed by a strong interaction between its diameter and length. For slender cavities with small diameters (e.g., 1 mm), increasing the tubular front cavity length effectively attenuates wind noise, particularly in the mid-to-high frequency range, by enhancing viscous and thermal boundary-layer losses along the internal wall. This passive damping mechanism functions as an acoustic low-pass filter. However, the efficacy of length extension diminishes sharply as the tubular front cavity diameter increases. For diameters of 2 mm and above, variations in tubular front cavity length produce negligible effects on the measured PSD, as wind noise generation becomes dominated by external flow–structure interaction at the cavity inlet rather than by internal propagation and dissipation. Importantly, this diameter-dependent transition from an internal-dissipation-dominated regime to an external-excitation-dominated regime was consistently observed in both controlled duct flow experiments and more realistic open-field anechoic chamber measurements, confirming it as an intrinsic property of the tubular front cavity geometry.

Although a measurement microphone was used in this study to ensure high-fidelity acquisition of intracavity pressure fluctuations, the results can be interpreted in the context of MEMS microphone performance. While the structural modifications yield a measurable reduction in flow-induced PSD, translating this directly to an equivalent SNR improvement is a hypothesis, not a demonstrated finding. Factors such as aperture diffraction and impedance mismatch will inherently introduce acoustic transmission losses. Therefore, statements regarding preserved frequency response and operational SNR enhancements are expected outcomes that necessitate dedicated electroacoustic validation in future work.

It should be noted that the present study focuses on simplified straight tubular cavities, which represent the most fundamental and idealized form of microphone front cavity geometry. While this study employed idealized straight tubular cavities to isolate fundamental mechanisms, real smartphone microphone inlets often incorporate protective meshes, acoustic resistors, and multi-layer channels. These elements may introduce additional acoustic impedance and alter the noise transmission characteristics. Nevertheless, the underlying principle—that internal dissipation dominates for small-diameter, elongated cavities—remains applicable. Future designs could integrate such cavities as pre-channels behind mesh covers, provided that the total volume does not exceed the available space and that acoustic transparency is maintained. The trade-off between wind noise suppression and low-frequency sensitivity loss should also be carefully evaluated in practical implementations. Future studies should extend this experimental framework to include direct measurements of microphone sensitivity and signal-to-noise ratio under simultaneous acoustic and flow excitation, thereby quantifying the net benefit of the proposed design in realistic operating conditions.

By elucidating the noise attenuation mechanisms and parameter sensitivities associated with this elementary geometry, the present work provides a foundational understanding that can guide the optimization of more complex front cavity designs. Future studies will extend this experimental framework to investigate composite and application-specific cavity geometries, with the ultimate goal of identifying optimal wind noise reduction strategies for miniature microphones in portable electronic devices.

## Data Availability

The original contributions presented in this study are included in the article. Further inquiries can be directed to the corresponding author.
